# Olfactory and taste disorders in COVID-19: a systematic review^☆^^☆☆^

**DOI:** 10.1016/j.bjorl.2020.05.008

**Published:** 2020-06-09

**Authors:** Klinger V.T. da Costa, Aline Tenório Lins Carnaúba, Katianne Wanderley Rocha, Kelly Cristina Lira de Andrade, Sonia M.S. Ferreira, Pedro de L. Menezes

**Affiliations:** a Universidade Federal de Alagoas (UFAL), Maceió, Alagoas, Brazil; b Centro Universitário Cesmac, Maceió, Alagoas, Brazil; c Universidade Estadual de Ciências da Saúde de Alagoas (UNCISAL), Maceió, Alagoas, Brazil

**Keywords:** COVID-19, SARS-CoV-2, Olfactory disorders, Taste disorders, Olfaction disorders, COVID-19, SARS-CoV-2, Distúrbios olfativos, Distúrbios do paladar, Distúrbios do olfato

## Abstract

**Introduction:**

The SARS-CoV-2 virus causes COVID-19, and it is responsible for the largest pandemic since the 1918 H1N1 influenza outbreak. The classic symptoms of the disease have been well defined by the World Health Organization; however, olfactory/gustatory disorders have been reported in some studies, but there are still several missing points in the understanding and in the consensus about the clinical management of these cases.

**Objective:**

To identify evidence in the scientific literature about olfactory/gustatory disorders, their clinical presentation, prevalence and possible specific treatments associated with COVID-19.

**Methods:**

A systematic review of articles published up to April 25, 2020 was performed in Medline, Cochrane Clinical Trials, ScienceDirect, Lilacs, Scopus and Google Schoolar, OpenGrey.eu, DissOnline, The New York Academy of Medicine and Reasearch Gate. Inclusion criteria: (1) Studies on patients with COVID-19; (2) Records of COVID-19 signs/symptoms, and olfactory/gustatory functions. Exclusion criteria: (1) Studies on non-human coronavirus; (2) Review articles; (3) Experimental studies (in animals or *in vitro*); (4) Olfactory/gustatory disorders initiated prior to SARS-CoV-2 infection. The risk assessment of bias of the selected studies was performed using the Newcastle-Ottawa scale.

**Results:**

Six articles from the 1788 records met the inclusion criteria and were analyzed. A total of 1457 patients of different ethnicities were assessed; of them, 885 (60.7%) and 822 (56.4%) had smell and taste disorders, respectively, with women being most often affected. There were olfactory/gustatory disorders even without nasal obstruction/rhinorrhea and beginning even before the signs/symptoms of COVID-19; the recovery of smell/taste, when it occurs, usually happened in the first two weeks after COVID-19 resolution. There is evidence that olfactory/gustatory disorders are strong predictors of infection by SARS-CoV-2, and it is possible to recommend patient isolation, as early as of the medical consultation, preventing the spread of the virus. No scientific evidence has been identified for effective treatments for any of the disorders.

**Conclusion:**

Olfactory/gustatory disorders may occur at varying intensities and prior to the general symptoms of COVID-19 and should be considered as part of the clinical features of COVID-19, even in mild cases. There is still no scientific evidence of specific treatments for such disorders in COVID-19 disease.

## Resumo

**Introdução:**

O vírus SARS-CoV-2 causa a COVID-19 e é responsável pela maior pandemia desde o surto de influenza H1N1 de 1918. Os sintomas clássicos da doença já foram bem definidos pela Organização Mundial da Saúde; entretanto, distúrbios olfativo-gustativos têm sido relatados em alguns estudos, mas ainda com várias lacunas no entendimento e no consenso sobre a condução clínica desses casos.

**Objetivo:**

Identificar evidências na literatura científica sobre os distúrbios olfativo-gustativos acerca da apresentação clínica, prevalência e possíveis tratamentos específicos associados à COVID-19.

**Método:**

Revisão sistemática de artigos publicados até 25 de abril de 2020 nas bases de dados: Medline, Cochrane Clinical Trials, ScienceDirect, Lilacs, Scopus e Google Schoolar, OpenGrey.eu, DissOnline, The New York Academy of Medicine e Reasearch Gate. Foram critérios de inclusão: 1) Estudos com indivíduos com COVID-19; 2) Registro dos sinais/sintomas da COVID-19 e das funções olfativo-gustativa. Foram critérios de exclusão: 1) Estudos sobre coronavírus não humano; 2) Artigos de revisão; 3) Estudos experimentais (em animais ou *in vitro*); 4) Distúrbios olfativos-gustativos iniciados previamente à infecção pelo SARS-CoV-2. A avaliação de risco de viés dos estudos selecionados foi feita por meio da escala de Newcastle-Ottawa.

**Resultados:**

Seis artigos dos 1.788 registros foram selecionados. Um total de 1.457 pacientes de diversas etnias foi avaliado; desses, 885 (60,7%) apresentaram perda do olfato e 822 (56,4%) perda do paladar, sendo as mulheres as mais afetadas. Os distúrbios olfativo-gustativos estiveram presentes mesmo sem obstrução nasal/rinorreia e com início mesmo antes dos sinais/sintomas clínicos da COVID-19; a recuperação do olfato/paladar, quando ocorre, geralmente se dá nas duas primeiras semanas após a resolução da doença. Há evidências que os distúrbios olfativo-gustativos sejam fortes preditores de infecção pelo SARS-CoV-2, podendo-se recomendar o isolamento do paciente, já a partir da consulta médica, para evitar a disseminação do vírus. Não foram identificadas evidências científicas para tratamentos eficazes para nenhum dos distúrbios.

**Conclusão:**

Podem ocorrer distúrbios olfativo-gustativos em intensidades variáveis e prévios aos sintomas gerais da COVID-19, devem ser considerados como parte dos sintomas da doença, mesmo em quadros leves. Não há ainda evidências científicas de tratamentos específicos para tais distúrbios na COVID-19.

## Introduction

Smell and taste are essential sensory functions both for good quality of life and for its preservation, by identifying several harmful odors and flavors. Viral Upper Respiratory Tract Infections (URTIs) can lead to Olfactory (OD) and Gustatory (GD) Disorders of varying degrees and duration,^1^ which can also occur in 70% of cases caused by rhinovirus, influenza virus and parainfluenza, respiratory syncytial virus, adenovirus and the severe acute respiratory syndrome virus (SARS-CoV-2).^2^

Coronavirus disease 2019 (COVID-19) became a viral pandemic that emerged from East Asia due to SARS-CoV-2.^3^ In Brazil, the number of cases continues to increase, with more than 66,000 confirmed cases by the end of April 2020^4^; however, it is estimated that the number is even higher, as the number of diagnostic tests performed is still insufficient for the national demand.

In COVID-19, the most common otorhinolaryngological symptoms are coughing, sore throat and dyspnea; rhinorrhea and nasal congestion have also been found; however, there are reports of OD/GD^5^, ^6^ and that they are present even before molecular confirmation of SARS-CoV-2 infection.^6^, ^7^ These observations were made recently, in countries that started to perform molecular tests for COVID-19 more extensively, in which patients infected with SARS-CoV-2 reported severe OD and GD without rhinorrhea or nasal obstruction. In the beginning, COVID-19 was not suspected in some of these patients, as they did not have fever, cough or any other classic general signs/symptoms of COVID-19. The OD and GD in COVID-19 still show gaps in their understanding of both the clinical spectrum and the treatment to be proposed.

The advancement of knowledge on a specific topic can be facilitated through systematic reviews (SR), which make it possible to identify, analyze and synthesize the best scientific evidence for best clinical practices, especially in the face of new clinical scenarios such as the current COVID-19 pandemic, in that randomized controlled studies and precise recommendations for their management are not yet available. The objective of this SR is to identify evidence of current knowledge on olfactory-gustatory disorders about the clinical presentation, prevalence and possible specific treatments associated with COVID-19.

## Methods

The creation of this Systematic Review (SR) aimed to answer the following question: “What is the association of olfactory/gustatory disorders, including their clinical presentations and treatments, with COVID-19?”, constructed with the help of the PICO strategy. This SR is in agreement with the “Preferred Reporting Items for Systematic Reviews and Meta-Analyses Statement (PRISMA)” checklist.^8^ The protocol was registered on April 24, 2020 at the International Prospective Register of Systematic Reviews – Prospero database (https://www.crd.york.ac.uk/PROSPERO/) under registration number CRD42020181524.

### Search strategy

The strategies aimed at a complete search, including descriptors (Medical Subject Headings - Mesh) and free terms, which consisted of: COVID-19, 2019-nCoV, 2019 novel coronavirus pneumonia, SARS-CoV, SARS-CoV-2, sars coronavirus, coronavirus, coronavirus infection, coronavirus COVID-19, novel coronavirus), COVID-19 clinical features, olfactory disorders, olfaction disorders and taste disorders. To identify the relevant articles for the proposed question, a search strategy was developed: (COVID-19 OR 2019-nCoV OR 2019 novel coronavirus pneumonia OR SARS-CoV OR SARS-CoV-2 OR sars coronavirus OR coronavirus OR coronavirus infection OR coronavirus COVID-19 OR novel coronavirus) AND (COVID 19 clinical features OR olfactory disorders OR olfaction disorders OR taste disorders); this strategy was adapted to the databases, and the corresponding terms were applied in Portuguese and Spanish at Lilacs database. There was no restriction on the language of publication. The complete search strategy is shown in the supplementary material (Appendix 1).

Between April 2 and 25, 2020, the following electronic databases were searched: Medline *via* PubMed, Cochrane Clinical Trials, ScienceDirect, Lilacs, Scopus and Google Schoolar, as well as the gray literature OpenGrey.eu, DissOnline, The New York Academy of Medicine and Reasearch Gate. To prevent citation bias, there was no manual search for the included articles and specialists in the field were not contacted.^9^

After the search, the references of each database were exported to the Mendeley® program (https://www.mendeley.com/) aiming to identify all articles in duplicate, promote greater selection reliability and proceed to the eligibility criteria step.

### Eligibility criteria

The following inclusion criteria were considered: (1) Observational studies with individuals diagnosed with coronavirus infection and (2) recording of the signs and symptoms of COVID-19, with clinical aspects related to olfactory and/or gustatory function. The following exclusion criteria were considered: (1) Studies on non-human coronavirus; (2) Review articles; (3) Experimental studies (in animals or *in vitro*); (4) Clinical report of OD/GD with onset prior to the SARS-CoV-2 infection.

### Data extraction

The search for the articles was carried out by two reviewers. The titles and abstracts of the retrieved articles were independently assessed by two researchers who were not blinded to the authors or the titles of journals. The full versions of the potentially eligible articles were retrieved for further evaluation and were carried out in three stages:

1. Identification and reading of titles in different electronic databases. Articles that clearly did not meet any of the inclusion or exclusion criteria were excluded.
2. Reading of the abstracts of the studies selected in the first phase. Likewise, articles that clearly did not meet any of the inclusion or exclusion criteria were excluded.
3. All studies that were not excluded in these first two stages were read in full for the selection of the ones that would be included in this Systematic Review (SR).

The main data for each article was fully collected and inserted into a database created in the Microsoft Office Excel 2010® software. There were no disagreements between the evaluators. A standard form for data storage was created based on the model adopted by Cochrane.^10^ For a better presentation of the results, it was decided to consider the following variables of the selected articles: Title, author, study location, study design, sample size (*N*), patients’ mean age and ethnicity, both the diagnostic method and the most prevalent general signs/symptoms and the severity of the clinical picture of COVID-19, methods used in the olfactory-gustatory evaluation and the prevalence of OD and GD. The outcomes sought in the studies were both the demographic data, prevalence and clinical expressions of OD/GD, as well as possible specific treatments for such disorders in patients diagnosed with COVID-19.

### Risk of bias assessment

The risk of bias was assessed according to the recommendations in the manual and the Newcastle-Ottawa scale adapted for cross-sectional observational studies.^11^ Study quality was independently assessed by two researchers and the divergences were evaluated by consensus. The maximum score to be achieved was 10 points and the items evaluated on the scale were: (1) Representativeness of the sample; (2) Sample size; (3) Characterization of the diagnosis; (4) Risk exposure assessment (risk factor); (5) Comparability, to investigate whether individuals in different groups of results are comparable, based on the study design or analysis, control of confounding factors; (6) Evaluation of results and (7) Statistical test (Table 1).

**Table 1:** The Newcastle-Ottawa scale adapted for cross-sectional observational studies.

**I selection: (maximum: 5 stars)**
*1. Representativeness of the sample*
(a) Truly representative of the mean in the target population^a^ (all subjects or randomized sample)
(b) A little representative of the mean in the target population^a^ (non-randomized sample)

*2. Selected user groups*
(a) Relevant selection of individuals to exclude factors that influence outcomes (such as certain diseases or medications that have a negative/positive effect on any condition)^a^

*3. Sample size*
(a) Justified and satisfactory (Power of calculation included)^a^

*4. Diagnosis*
(a) Characterization of the diagnosis^b^
(b) Determination of exposure^a^

**II comparability (maximum: 2 stars)**
*1. The subjects in different groups of results are comparable, based on the study design or analysis. Confounding factors are controlled*
(a) The study controls the most important factor (select one)^a^
(b) Control of the study for any additional factors^a^

**III results (maximum: 3 stars)**
*1. Determination of the method*
(a) Validated measurement method^b^
(b) Measurement method not validated, but the method is available or described^a^

*2. Statistical tests*
(a) The statistical test used to analyze the data is clearly described and appropriate, and the measurement of the association is shown; including SD/SE and the probability level (*p*-value)^a^

**Total (maximum: 10 stars)**

After these evaluations, the selected studies were submitted to a statistical analysis to verify the possibility of building a meta-analysis. This analysis combines and summarizes the results of several studies, thus increasing the accuracy and power of evidence of the results.

## Results

### Included studies

**Figure 1:**
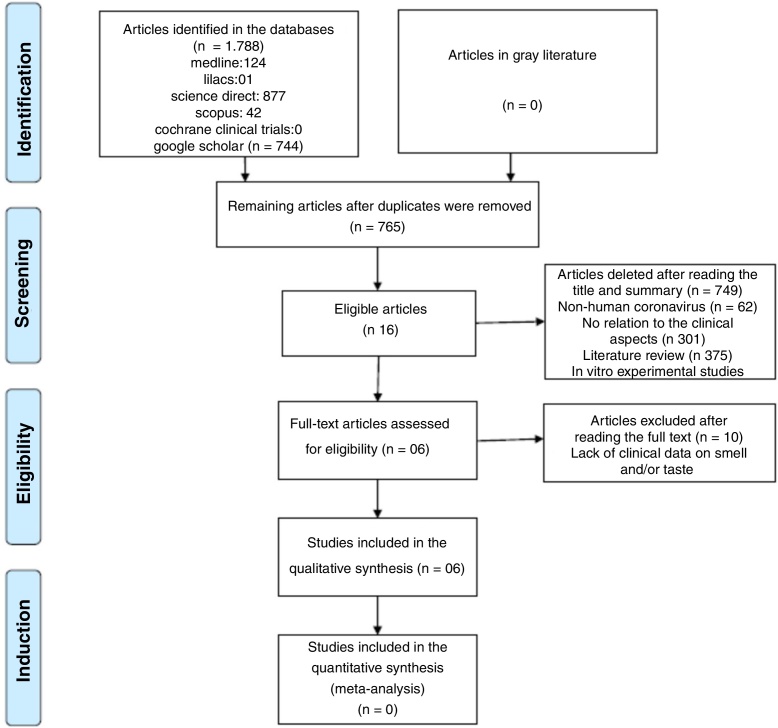
Diagram of identification and selection flow.

In the first phase of this SR, 1788 articles were found in six databases and none in the gray literature. After eliminating 1023 studies in duplicate, 765 were selected for the reading of titles and abstracts. A total of 749 were excluded according to the established selection criteria (62 studies on non-human coronavirus, 301 due to lack of data on clinical aspects, 375 literature reviews and 11 experimental studies *in vitro*) and 16 articles were selected to be read in full. After the reading, 10 articles were excluded as they did not have data on smell/taste (Table 2). Therefore, six full articles were included in the qualitative analysis (Table 3). The entire article selection process is described in Fig. 1, which shows the Prisma flow diagram for inclusion.

**Table 2:** Full texts excluded from the analysis.

Author	Location	Year	Reason for exclusion
Chen et al.	China	2020	Absence of data on smell/taste
Cheng et al.	China	2019	Absence of data on smell/taste
Huang et al.	China	2020	Absence of data on smell/taste
Jin et al.	China	2020	Absence of data on smell/taste
Piva et al.	USA	2020	Absence of data on smell/taste
Tian et al.	China	2020	Absence of data on smell/taste
Wang et al.	China	2020	Absence of data on smell/taste
Young et al.	Singapore	2020	Absence of data on smell/taste
Zheng et al.	China	2019	Absence of data on smell/taste
Zhu et al.	China	2020	Absence of data on smell/taste

**Table 3:** Studies selected according to the inclusion and exclusion criteria established in the systematic review.

Article	Title	Author	Location	Study design	*n*
1	*Neurological Manifestations of Hospitalized Patients with* COVID-19 *in Wuhan, China: a retrospective case series study*	Mao et al.^12^	China	Retrospective	214
2	*Olfactory and gustatory dysfunctions as a clinical presentation of mild-to-moderate forms of the coronavirus disease* (COVID-19): *a multicenter European study*	Lechien et al.^13^	Belgium, France, Spain and Italy	Multicentric (transversal)	417
3	*Smell Dysfunction: A Biomarker for* COVID-19	Moein et al.^14^	Iran	Cross-sectional, observational	60
4	*Association of Chemosensory Dysfunction and* COVID-19 *in Patients Presenting with Influenza-like Symptoms*	Yan et al.^15^	USA	Cross-sectional, observational	59
5	*Self-reported olfactory loss associates with outpatient clinical course in* COVID-19	Yan et al.^16^	USA	Retrospective	128
6	*Loss of smell and taste in combination with other symptoms is a Strong predictor of* COVID-19 *infection*	Menni et al.^17^	United Kingdom	*Survey*	579
*Total*					1457

### Risk of bias assessment

The analysis of the quality of the included articles and, consequently, of the risk of bias, is shown in Table 4. All included studies are characterized as observational and cross-sectional studies. Moreover, in the final evaluation, all obtained a percentage of quality equal to or greater than 80% (8/10), and four of the six articles obtained the maximum score of 90% (9/10).

**Table 4:** Quality assessment of cross-sectional studies using the Newcastle-Ottawa Scale (adapted).

Article	Selection^a^				Comparability (control of the most important factor)^b^	Result^c^		Final evaluation^d^
	Sample representativeness	Relevant selection	Sample size	Determination of exposure/diagnosis		Validated measurement method	Statistical test	
Mao et al.^12^	1	1	0	3	1	2	1	9
Lechien et al.^13^	1	1	0	3	1	2	1	9
Moein et al.^14^	1	0	0	3	1	2	1	8
Yan^a^ et al.^15^	1	0	0	3	1	2	1	8
Yan^b^ et al.^16^	1	1	0	3	1	2	1	9
Menni et al.^17^	1	1	1	3	1	2	1	9

a Maximum score (5 points).

b Maximum score (2 points).

c Maximum score (3 points).

d Maximum score (10 points).

Only one of the studies carried out a census study, the other studies did not perform a random selection of patients, which compromises the representativeness of the samples, but took into account the sample size.

All studies were concerned with the participants’ selection, defining the inclusion and exclusion criteria, in order to exclude factors that could influence the results found.

The size of the studied samples was adequate to the central limit theorem for all studies, and only two studies had samples smaller than 300 subjects.

The characterization of the diagnosis and the determination of exposure were very well described in all studies. All studies controlled at least one variable, allowing an efficient comparison among all studies.

The evaluation of the results was carried out in all studies through a specific report and the methods were described and validated in all studies.

Finally, all studies showed appropriate statistical tests, with description of the statistical tests, the levels of significance and the applications used.

It was not possible to perform a meta-analysis in this SR because the articles included had very different methodologies and measures resulting from the tests.

### Data analysis

Regarding the overall characteristics of the included articles (Table 3), the six studies were published in 2020, one in China,^12^ one in Europe,^13^ one in Iran,^14^ two in the United States^15^, ^16^ and one in the United Kingdom.^17^ The Chinese study (Mao et al.)^12^ retrospectively evaluated 214 patients from three hospitals in Wuhan; the European study (Lechien et al.)^13^ cross-sectionally evaluated 417 patients in a multicentric model; the Iranian study (Moein et al.)^14^ studied 60 patients with COVID-19 and 60 subjects in the control group, matched for age and gender; the two North-American studies were carried out by the same group of researchers (Yan et al.)^15^, ^16^; while the British study (Menni et al.)^17^ evaluated, through a census study, 579 COVID-19 positive and 1123 COVID-19 negative patients. The demographic data, general symptoms and the prevalence of OD and GD in patients in the six studies are depicted in Table 5.

**Table 5:** Demographic data, overall clinical data, olfactory-gustatory diagnostic method, prevalence of olfactory and gustatory disorders in patients in the three selected studies.

Article	Author	*N*: *n* (%)	Age (±SD)	Ethnicity, *n* (%)	Diagnosis of COVID-19	Severity of COVID-19, *n* (%)	Overall symptoms/signs in COVID-19, *n* (%)	Olfactory/gustatory evaluation method	Prevalence, *n* (%)
1	Mao et al.^12^	214: 127 (59.3)♀; 87 (40.7)♂	52.7 (±15.5) years	Asian 214 (100)	RT-PCR^a^	Severe 88 (41.1)	Fever 132 (61.7)	Analysis of clinical records in patients’ files	Gustatory disorder 12 (5.6)
							Dry cough 107 (50)		
						Mild-moderate 126 (58.9)	Anorexia 68 (31.8)		Olfactory disorder 11 (5.1)
2	Lechien et al.^13^	417: 263 (63.1)♀; 154 (36.9)♂	36.9 (±11.4) years	Europeans 389 (93.3)	RT-PCR^a^	Mild-moderate 417 (100)	Dry cough 325 (78)	sQOD-NS^b^	Olfactory disorder 357 (85.6)
				African descendants 15 (3.6)			Myalgia 241 (58)		
				South-American 11 (2.7)			Anorexia 216 (52)		Gustatory disorder 342 (82)
				North-American 1 (0.2)			Fever 200 (48)		
				Asian 1 (0.2)					
3	Moein et al.^14^	60: 20 (33.3)♀; 40 (66.6)♂ 60 (control)	46.55 (±12.17) years	Asian 60 (100)	RT-PCR^a^	Mild 25(42)	Fever 46 (77)	UPSIT^c^	Olfactory disorder 59 (98.3)
						Moderate 29 (48)	Dry cough 35 (58)		
						Severe 6 (10)	Shortness of breath 31 (52)		Gustatory disorder 14 (24)
4	Yan et al.^15^	58: 38 (64.4)♂; 20 (35.6)♀; 203 COVID-19 negatives	<50 years: 38 (65.5)	North-Americans 58 (100)	RT-PCR^a^	Mild 54 (93.1)	Fatigue 48 (81.4)	Questionnaire	Gustatory disorder 41 (71.2)
			>50 years: 21 (34.5)			Moderate/severe 4 (6.9)	Fever 41 (69.5)		Olfactory disorder 40 (67.8)
5	Yan et al.^16^	128: 67 (52.3)♀; 61 (47.7)♂	48.25 (±16.75) years	North-Americans 8 (30.8)	RT-PCR^a^	Mild 102 (79.7)	Dry cough 112 (87.5)	Medical record, e-mail and telephone	Olfactory disorder 75 (58.6%)
				African descendants 3 (11.5)			Fever 90 (70.3)		
				South-American 7 (26.9)		Moderate/severe 26 (20.3)	Fatigue 90 (70.3)		Gustatory disorder 70 (54.6%)
				Asian 4 (15.4)					
				Others 4 (15.4)					
6	Menni ey al.^17^	579: 400 (69)♀; 179 (31)♂; 1123 negative for COVID-19	40.79 (±11.84) years	Not evaluated	RT-PCR^a^	Mild 579 (100)	Fatigue 458 (80.1)	RADAR COVID-19 Application	Olfactory/gustatory disorder 343 (59.4)
							Dry cough 335 (58)		
							Shortness of breath: 194 (49.4)		

♂, male; ♀, female; SD, standard deviation.

a Real-time reverse transcription polymerase chain reaction.

b The smell and taste component of the National Health and Nutrition examination Survey (simplified version of the Questionnaire of Olfactory Disorders-Negative Statements).

c The University of Pennsylvania Smell Identification Test.

In total, the six studies evaluated 1457 patients (Table 3). In the study by Mao et al.,^12^ the sample consisted only of Chinese patients; in the study by Lechien et al.,^13^ most of the sample consisted of European patients; in the study by Moein et al.,^14^ all patients were Iranians; in the two studies by Yann et al.,^15^, ^16^ most patients were North-American; and in the study by Menni et al.,^17^ the ethnicity of the study participants was not recorded.

The exam used for diagnosing COVID-19 in the six studies was the RT-PCR (Real-Time Reverse-Transcription Polymerase Chain Reaction). Regarding the most prevalent overall signs/symptoms of COVID-19, there were differences between groups; fever was the most prevalent sign in the studies by Mao et al.^12^ and by Moein et al.^14^; fatigue was the first symptom in the first study by Yan et al.^15^ and of Menni et al.^17^; coughing was the first sign in the second study by Yan et al.^16^ and in the study by Lechien et al.^13^ (Table 5).

Mao et al.^12^ carried out the study at three hospitals designated for the care of patients with COVID-19, which belonged to the Union Hospital of Huazhong University of Science and Technology in Wuhan, China. Clinical data were extracted from electronic medical records and reviewed by a trained medical team. Neurological symptoms fall into three categories: skeletal muscle symptoms, symptoms or diseases of the Central Nervous System (CNS) and Peripheral Nervous System (PNS) symptoms, including OD and GD. Data on all neurological symptoms were verified by two trained neurologists. Of the 214 patients studied, 126 (58.9%) had mild-moderate disease, 127 (59.3%) were females and the mean age of the group was 52.7 ± 15.5 years. Compared to the patients with mild-moderate disease, critically-ill patients were older (58.7 ± 15.0 *vs.* 48.9 ± 14.7 years) and showed fewer typical symptoms, such as fever in 40 (45.5%) *vs*. 92 (73%) and coughing in 30 (34.1%) *vs*. 77 (61.1%). GD was identified in 12 (5.6%), and OD in 11 (5.1%) patients; regarding these disorders, there were only references to hypogeusia and hyposmia and they were the main symptoms reported by patients with PNS alterations, with 12/19 (63%), 11/19 (57.8%); respectively. There were no records regarding the onset of olfactory/gustatory disorders, duration, resolution or specific treatments.

Lechien et al.^13^ carried out the multicentric European study through the COVID-19 Task Force of the Young-Otolaryngologists of the International Federations of Oto-rhino-laryngological Societies (YO-IFOS) in six Belgian, three Italian and two French hospitals. Patients with olfactory-gustatory disorders of which onset occurred prior to the pandemic, patients without laboratory diagnosis of COVID-19, patients who were in the intensive care unit at the time of the study (due to their health status) were excluded. Therefore, the sample consisted only of patients with mild to moderate disease. Clinical data were collected prospectively during the otorhinolaryngological consultation or by telephone for patients undergoing home treatment. In this study, patients filled out a questionnaire based on “The smell and taste component of the National Health and Nutrition Examination Survey” and the simplified version of the “Questionnaire of Olfactory Disorders-Negative Statements” (sQOD-NS).^18^ Of the 417 patients, 389 (93.3%) were European, 263 (63.1%) were females, all with mild to moderate pictures of COVID-19 and with a mean age of 36.9 ± 11.4 years. In the evaluated sample, 85.6% and 88.8% of the patients reported OD and GD, respectively; there was a significant association between the two disorders (*p* < 0.001). Of the 357 patients with OD, 284 (79.6%) had anosmia, 73 (20.4%) had hyposmia, 45 (12.6%) had phantosmia and 115 (32.4%) had parosmia. OD appeared before, during or after the other signs/symptoms of COVID-19 in 11.8%, 22.8% and 65.4% of cases, respectively. Among patients that had already recovered from COVID-19, 63% of them remained with OD. Among the patients who recovered their sense of smell, 33%, 39.6%, 24.2% and 3.3% did so in the periods of 1–4, 5–8, 9–14 and more than 15 days of resolution of the COVID-19 picture, respectively. In anosmia cases, olfactory function normalized over the first eight days after disease resolution in 67.8% of cases. An association was observed between fever and anosmia (*p* = 0.014). Women were significantly more affected by OD (with both hyposmia and anosmia) and GD than men (*p* = 0.001). Regarding the treatment for OD, saline nasal lavage, nasal corticosteroids and oral corticosteroids were used by 16.7%, 8.1% and 2.5% of the patients, respectively; GDs were treated in 1.4% of cases with l-carnitine or trace elements associated with vitamins. No results were observed regarding the effectiveness of such specific treatments for OD and GD.

Moein et al.^14^ carried out the study with 60 SARS-CoV-2 positive patients who were admitted to Masih Daneshvari Hospital in Tehran, Iran. At the time of the olfactory test, everyone was hospitalized during the recovery period of the disease. A control group was created with 60 healthy subjects, paired by gender and age with the 60 patients with the infection. The Persian version of the University of Pennsylvania Smell Identification Test (UPSIT), with 40 odors, was used in this study (Sensonics International, Haddon Hts., NJ, USA) with the help of a trained examiner; the test provides an absolute dysfunction index (anosmia, severe hyposmia, moderate hyposmia, mild hyposmia, normosmia, faking), as well as the relative dysfunction based on normative percentile classifications adjusted for age and gender. Of the 60 patients studied with COVID-19, 25 (42%) had mild, 29 (48%) had moderate and 6 (10%) had severe disease; 40 (66.6%) were males and the mean age of the group was 46.5 ± 12.1 years. Of the 60 patients, OD was identified in 59 (98.3%) and GD in 14 (24%), in contrast to the control group, that showed OD in 18% of the individuals; of the 60 patients, 15 (25%) had anosmia, 20 (33%) had severe hyposmia, 16 (27%) had moderate hyposmia, 8 (13%) had mild hyposmia, and 1 (0.2%) was normal. The 59 patients with OD reported that the disorder started at the same time or immediately after the onset of the signs/symptoms of COVID-19. There were no detailed data on the type of gustatory disorder or specific treatments for OD/GD in the studied sample.

In their first study, Yan et al.^15^ evaluated 58 patients at the University of California San Diego Health hospital with positive laboratory tests for COVID-19 and 203 negative tests. The ODs and GDs were assessed with the help of a questionnaire using a platform (Qualtrics®). The questions were based on a 10-point continuous bar for the olfactory function, meaning: 0 (anosmia), 10 (normal smell), and a 10-point bar for the gustatory function, meaning: 0 (ageusia), 10 (normal gustatory function); the lower the score, the greater the severity of OD and GD. Of the 58 studied patients with COVID-19, 54 (93.1%) had mild disease, 29 (50%) were males and 38 (64.4%) were under 50 years of age. Loss of smell and taste was reported in 40 (68%) and 42 (71%) of COVID-19 positive individuals, respectively. In 12 (22%) patients, OD was already present at the beginning of the condition. The smell and taste function impairment was independently and strongly associated with COVID-19 positivity (Anosmia: Adjusted Odds Ratio – Aor = 10.9; 95%CI: 5.08–23.5; Ageusia: Aor = 10.2; 95%CI: 4.74–22.1). Compared to other symptoms of COVID-19 infection, loss of smell and taste showed the greatest magnitudes of association with COVID-19 positivity (Anosmia: OR = 10.9; 95%CI: 5.6–21, 0; Ageusia: OR = 11.9; 95%CI: 6.1–23.2). Of the patients who reported OD, 43 (73.6%) reported anosmia resolution at different intervals after recovery from COVID-19 (18.4% in the first week; 36.8% in the second week, and 18.4% in the third or fourth week); 15 (26.4%) of the cases did not show olfaction recovery. No results were observed regarding the resolution of the GD condition or specific treatments for both disorders.

In their second study, Yan et al.^16^ retrospectively evaluated 128 patients with a positive laboratory test for COVID-19 recorded in the San Diego Hospital system. Smell and taste were self-assessed in relation to the period of the disease and were compared to the levels before the disease (loss of smell/taste *vs.* normal function); at first, these data were extracted from patients’ medical records; when absent, the interview was performed *via* email or telephone. Of the 128 patients with COVID-19, 102 (79.7%) had mild disease, 67 (52.3%) were females and the mean age was 48.25 ± 16.75 years. Of the 128 patients, 75 (58.6%) had OD and 70 (54.6%) had GD. Patients who reported loss of smell were ten times less likely to be hospitalized due to COVID-19 (OR = 0.09; 95%CI: 0.01–0.74) when compared to those without loss of smell. ODs were not associated with any other measure typically related to the decision to admit the patient, suggesting that loss of smell is truly a factor that can be used as a marker for milder manifestations of COVID-19. Thus, the authors suggest that OD may be a clinical marker to help stratify disease severity at the beginning of SARS-CoV-2 infection. There was no detailed data on the type of GD present or specific treatments for any of the disorders in the studied sample.

Menni et al.^17^ performed a census study using the RADAR COVID-19 application with 579 patients who tested positive for SARS-CoV-2 in the UK and 1123 negative ones. Of the 579 patients, all had mild disease, 400 (69%) were females and the mean age was 40.79 ± 11.84 years. OD and GD were present in 341 (59%) patients positive for COVID-19, compared to 202 (18%) that tested negative for the disease, resulting in an odds ratio (OR) of COVID-19 diagnosis (95%CI = 6.59 [5.25; 8.27], *p* = 1.90 × 10–59). The combination of loss of smell and taste, fever, persistent cough, fatigue, diarrhea, abdominal pain and loss of appetite was predictive for a positive COVID-19 test, with a sensitivity of 0.54 (0.44; 0.63); specificity of 0.86 (0.80; 0.90): ROC-AUC = 0.77 (0.72; 0.82) in the set of tests and ROC-AUC cross-validation = 0.75 (0.72; 0.77). Overall, without adjustments, loss of smell and taste had a positive predictive value of 61.7%. The results suggest that loss of taste/smell is a strong predictor of SARS-CoV-2 infection. There were no detailed data on the subtype of OD and GD, onset and resolution in relation to the COVID-19 severity, or specific treatments for any of the disorders.

## Discussion

The current SR was designed to identify evidence in the scientific literature about both olfactory/gustatory disorders in relation to the clinical presentation, prevalence and possible specific treatments associated with COVID-19.

The six studies showed different prevalence rates for OD and GD. The study with the greatest divergence from the others regarding the prevalence of OD and GD was the Chinese study by Mao et al.,^12^ in which the prevalence of OD was 19 times lower than in the study by Moein et al.^14^ and 14 times lower than in the study by Lechien et al.^13^ These differences can be explained by two possibilities: (1) The study models, because while the Chinese study, with lower prevalence rates of OD and GD, was based only on data from medical records in which gustatory/olfactory complaints may have been neglected, while the other studies used tools aimed at assessing smell and taste; (2) Chinese patients, in fact, had few olfactory-gustatory complaints.

We observed that in the Chinese, Iranian and North-American studies, the mean age was older than in European studies; such divergence can be explained by the sample composition regarding the severity of COVID-19, since the Asian and North-American studies had patients with the severe form of the disease, more common in older patients and those with comorbidities,^19^, ^20^, ^21^, ^22^ while in the European groups there were only mild to moderate cases.

The clinical behavior of OD and GD varied in several aspects: it was observed that anosmia was the most prevalent OD and its onset can occur even before the symptoms/signs of COVID-19. There is evidence that loss of smell may not only be a clinical marker for SARS-CoV-2 infection but can also help to stratify the degree of illness at the onset of infection. Considering the difficulties in terms of public health for the performance and acquisition of results in a timely manner by RT-PCR, the evaluation of these disorders in times of COVID-19 pandemic can be useful to guide immediate isolation, even before having access to the test result, favoring the prevention of the dissemination of the virus. A opposed to OD that occurs with other viral infections, OD in most patients with COVID-19 was not associated with nasal obstruction or rhinorrhea. OD recovery occurs, in most cases, within the first two weeks, while only 3.3% manage to recover their sense of smell/taste after this period and it is not yet known whether the others who persist with OD can recover their sense of smell in the long term. The olfactory neuroepithelium has considerable regenerative capacity, leading to spontaneous improvement of smell, at varying degrees, over time^23^ if the stem cell layer is not markedly damaged.^24^, ^25^, ^26^ Regarding the GD, detailed information was not found; however, the association of GDs with ODs was significant, and the recovery of taste depended on the recovery of the smell.

The variability of the clinical spectrum of OD/GD in COVID-19 is broad and certainly defined by biological characteristics, viral, cellular and molecular, of patients that vary according to ethnicity. Of the six assessed studies, the two studies that did not include severe forms of COVID-19 consisted of European patients only; however, they maintained high prevalence rates of OD and GD. Mutations in surface proteins (Spike protein (S) and Nucleocapsid protein (N) of SARS-CoV-2 in different regions of China have already been observed,^27^ and can modify the biological behavior of the virus in relation to tissue affinity.^20^ The Angiotensin-Converting Enzyme 2 (ACE2) acts as a receptor for SARS-CoV-2, especially in the olfactory bulb,^28^ so that some of its variants observed in different ethnicities could reduce its ability to receive the virus S protein by modifying its susceptibility, symptoms and clinical outcomes^29^; there are relatively high expressions of ACE2 and transmembrane serine protease 2 (TMPRSS2) in the human olfactory epithelium^30^; the non-neuronal expression of ACE2/TMPRSS2 may possibly make the olfactory epithelium a reservoir of viruses.^30^ The expression of TMPRSS2 seems to be higher compared to that of ACE2 and occurs in both neurons and non-neuronal cells in the olfactory epithelium and with a mosaic pattern, suggesting that some olfactory neurons may be more vulnerable to viral infection.^31^, ^32^

Regarding specific treatments, there was no evidence of specific drugs that were effective for the recovery of olfactory/gustatory functions. Nasal lavage was predominant in the studied groups.

Therefore, patients with COVID-19 of different ethnicities were evaluated, with high prevalence of impaired smell/taste, with women being the most significantly affected gender; unlike other viral etiologies, SARS-CoV-2 can lead to loss of olfactory/gustatory functions even without the presence of nasal obstruction/rhinorrhea and with the onset of OD/GD even before the clinical signs/symptoms of COVID-19. There is evidence that OD/GD are strong predictors of infection by SARS-CoV-2, and it is possible to recommend the isolation of the patient as of the medical consultation and still without the result of the laboratory test, to prevent the dissemination of the virus. The recovery of smell/taste, when it occurs, usually does so within the first two weeks after the resolution of COVID-19. No scientific evidence has been identified for effective treatments for any of the disorders.

## Conclusion

The studies have shown that both olfactory and gustatory disorders of varying intensities and in which onset occurred prior to the general symptoms of SARS-CoV-2 infection may occur, and such disorders should be considered as part of the symptoms that may be present, even in mild cases of COVID-19. There is still no scientific evidence of specific treatments for such disorders in COVID-19.

## Conflicts of interest

The authors declare no conflicts of interest.

## Figures and Tables

**Table:** 

**Medlinevia Pubmed**
(COVID-19 OR 2019-nCoV OR 2019 *novel coronavirus pneumonia* OR SARS-CoV OR SARS-CoV-2 OR *sars coronavirus* OR *coronavirus* OR *coronavirus infection* OR *coronavirus* COVID-19 OR *novel coronavirus*) AND (COVID-19 *clinical features* OR *olfactory disorders* OR *olfaction disorders* OR *taste disorders*)
**LILACS**
(COVID-19 OR 2019*-nCoV* OR 2019 *novel coronavirus pneumonia* OR SARS-CoV OR SARS-CoV-2 OR *sars coronavirus* OR *coronavirus* OR *coronavirus infection* OR *coronavirus* COVID-19 OR *novel coronavirus*) AND (COVID-19 *clinical features* OR *olfactory disorders* OR *olfaction disorders* OR *taste disorders*)
**Science Direct**
(COVID-19 OR *2*019*-nCoV* OR 2019 *novel coronavirus pneumonia* OR SARS-CoV OR SARS-CoV-2 OR *sars coronavirus* OR *coronavirus* OR *coronavirus infection* OR *coronavirus* COVID-19 OR *novel coronavirus*) AND (COVID-19 *clinical features* OR *olfactory disorders* OR *olfaction disorders* OR *taste disorders*)
FILTERS: (YEAR 2019–2020), (TYPE OF ARTICLE: ORIGINAL)
**SCOPUS**
(COVID-19 OR 2019*-nCoV* OR 2019 *novel coronavirus pneumonia* OR SARS-CoV OR SARS-CoV-2 OR *sars coronavirus* OR *coronavirus* OR *coronavirus infection* OR *coronavirus* COVID-19 OR *novel coronavirus*) AND (COVID-19 *clinical features* OR *olfactory disorders* OR *olfaction disorders* OR *taste disorders*)
**COCHRANE CLINICALTRIALS**
(COVID-19 OR 2019*-nCoV* OR 2019 *novel coronavirus pneumonia* OR SARS-CoV OR SARS-CoV-2 OR *sars coronavirus* OR *coronavirus* OR *coronavirus infection* OR *coronavirus* COVID-19 OR *novel coronavirus*) AND (COVID-19 *clinical features* OR *olfactory disorders* OR *olfaction disorders* OR *taste disorders*)
**GOOGLE SCHOLAR**
(COVID-19 OR 2019*-nCoV* OR 2019 *novel coronavirus pneumonia* OR SARS-CoV OR SARS-CoV-2 OR *sars coronavirus* OR *coronavirus* OR *coronavirus infection* OR *coronavirus* COVID-19 OR *novel coronavirus*) AND (COVID-19 *clinical features* OR *olfactory disorders* OR *olfaction disorders* OR *taste disorders*)
